# Design and Analysis of the Elastic-Beam Delaying Mechanism in a Micro-Electro-Mechanical Systems Device

**DOI:** 10.3390/mi9110567

**Published:** 2018-11-02

**Authors:** Fufu Wang, Lu Zhang, Long Li, Zhihong Qiao, Qian Cao

**Affiliations:** 1 Key Laboratory of Space Utilization, Technology and Engineering Center for space Utilization, Chinese Academy of Sciences, Beijing 100094, China; wangfufu2004@sina.com (F.W.); qiaozhihong@csu.ac.cn (Z.Q.); caoqian@csu.ac.cn (Q.C.); 2 Qian Xuesen Laboratory of Space Technology, NO. 104 Youyi Road, Haidian District, Beijing 100094, China; lilong@qxslab.cn

**Keywords:** micro-electro-mechanical systems (MEMS), delaying mechanism, safety and arming system

## Abstract

The delaying mechanism is an important part of micro-electro-mechanical systems (MEMS) devices. However, very few mechanical delaying mechanisms are available. In this paper, an elastic-beam delaying mechanism has been proposed innovatively through establishing a three-dimensional model of an elastic-beam delay mechanism, establishing the force and the parameters of an elastic-beam delay mechanism, deriving the mathematical model according to the rigid dynamic mechanics theory, establishing the finite element model by using Ls-dyna solver of the Ansys software, and carrying out the centrifugal test. Simulation and test results match theoretical results quite well. It is believed that the elastic-beam delaying mechanism is quite effective and useful to slow the speed of the movable part in MEMS devices.

## 1. Introduction

The function of the delaying mechanism is to delay the appropriate time to ensure that the mechanism completes the corresponding action. The traditional delaying mechanism mainly includes the non-returning clock mechanism, the gas or liquid damping mechanism, the quasi-fluid mechanism, and so on [1,2].

Micro-electro-mechanical systems (MEMS) are a relatively new and fast-growing field in microelectronics. Micro-electro-mechanical systems are commonly used as actuators, sensors, and radio frequency and microfluidic components, as well as bio-composites, with a wide variety of applications in health care, automotive, and military industries. It is expected that the market for MEMS will grow to over $30 B by 2050 [3,4,5,6,7].

With the rapid development of MEMS technology, the demand for miniaturization of delaying mechanisms has become more urgent. At present, there are a large number of MEMS delaying mechanism research, which are mainly divided into two categories. One category is the delaying mechanism based on electricity [8,9,10,11,12,13,14,15,16], which has high-control precision; Dalian University of Technology in China has studied the v-shaped micro-electric-thermal delaying mechanism based on MEMS technology [8], and the university of Toulouse in France has studied MEMS electro-powder delaying mechanism [9]. The other category is the mechanical delaying mechanism [17]; KAMAN INC. of the United States demonstrated a MEMS mechanical delaying mechanism and its delay action does not require electronic control, and it is free from electromagnetic interference and high reliability, and it is particularly suitable for battlefield use in complex electromagnetic environments.

**Figure 1:**
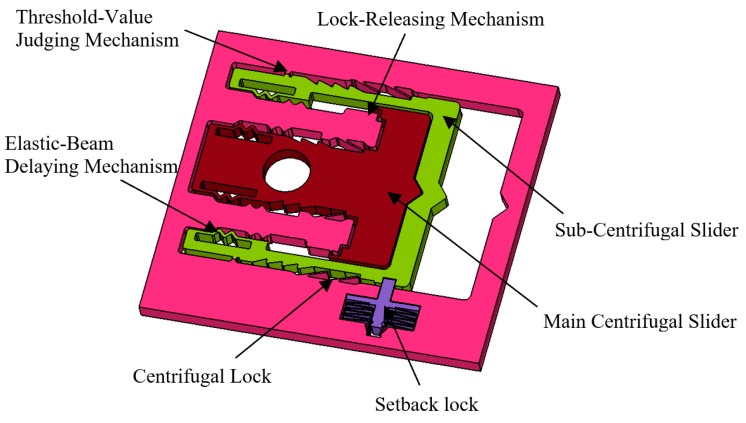
The mechanical MEMS S&A system.

**Figure 2:**
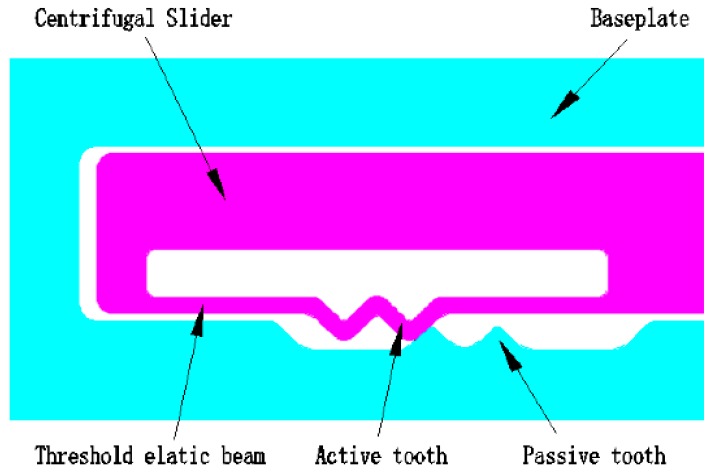
The elastic-beam delaying mechanism.

In this paper, the MEMS Safety and Arming (S&A) system used in small-caliber ammunition is proposed innovatively, and the concrete structure as shown in Figure 1. The size of the MEMS S&A system is 10 mm × 13 mm × 0.5 mm. The MEMS S&A system is composed of threshold-value judging mechanism, lock-releasing mechanism, elastic-beam delaying mechanism, centrifugal lock, setback lock, sub-centrifugal slider, and main centrifugal slider. It has two functions [18,19,20,21,22,23,24,25,26,27,28]: one function is that after the ammunition goes out of the antiaircraft gun, it needs a certain time delay to ensure that the main centrifugal slider does not move to the designated position, so as to ensure that the ammunition does not explode at the muzzle or launch area, which can guarantee the safety of the ammunition. The other function is that when ammunition arrives at the designated area, it needs the main centrifugal slider to move to the designated position to ensure the reliable function of ammunition. The elastic-beam delaying mechanism plays an important role in ensuring the safety of ammunition. Figure 2 shows that the elastic-beam delaying mechanism is composed of baseplate, centrifugal slider, passive tooth, active tooth, and threshold elastic beam, which can slow the speed of the movable part to ensure a proper delay time and guarantee the safety of the ammunition.

## 2. Model and Theoretical Analysis

### 2.1. Model

**Figure 3:**
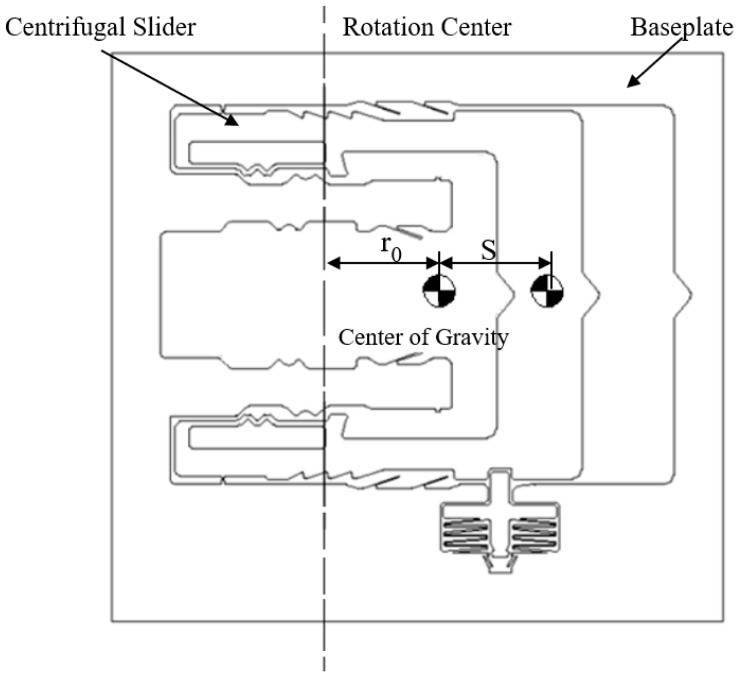
The center of gravity, rotation center, and stroke of the centrifugal slider.

**Figure 4:**
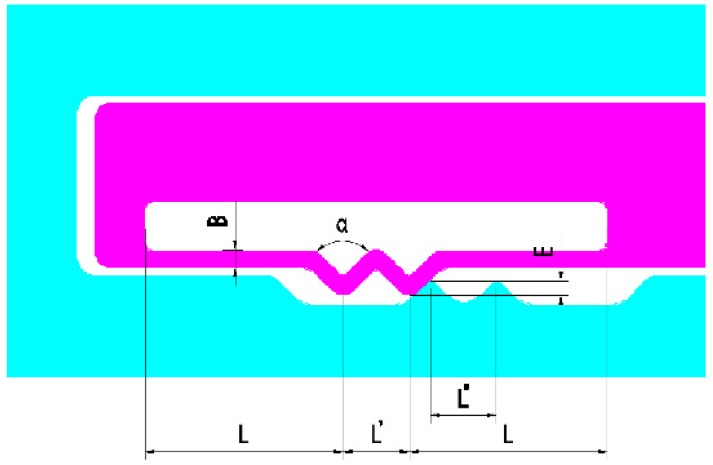
The parameters of elastic-beam delaying mechanism.

**Figure 5:**
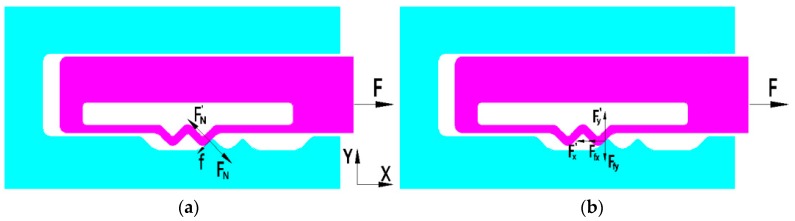
The force of elastic-beam delaying mechanism: (**a**) overall view, (**b**) decomposition view.

The center of gravity, rotation center, and stroke of the sub-centrifugal slider are shown in Figure 3. The parameters of elastic-beam delaying mechanism are shown in Figure 4. The force of elastic-beam delaying mechanism are shown in Figure 5. Based on Figure 4, the main variables are parameterized, shown as Table 1.

### 2.2. Force Analysis

We have some hypotheses:

- Neglecting the factors such as friction and air resistance;
- The process of active tooth contact passive tooth movement is from uniform acceleration to uniform deceleration.

According to the Figure 4 and Figure 5, we can get the centrifugal force of centrifugal slider

(1) $$F=mr\omega^{2}=mr{\left(\frac{2\pi n}{60}\right)}^{2}$$

where, *m* is the mass of centrifugal slider; *r* is the eccentricity of centrifugal slider; *n* is the rotating speed of projectile.

According to the Figure 4 and Figure 5b, we can get

(2) {Fx′=FN′cos(α2)=FN(w)cos(α2)Fy′=FN′sin(α2)=FN(w)sin(α2)

(3) {Ffx=fsin(α2)=μFN(w)sin(α2)Ffy=fcos(α2)=μFN(w)cos(α2)

where, $F_{N}(w)$ is the pressure of the active tooth to the passive tooth; μ is the friction coefficient of the active tooth to the passive tooth; *f* is the friction of the active tooth to the passive tooth; ${F_{x}}^{\prime }$, $F_{y}{}^{\prime }$, $F_{fx}$, $F_{fy}$ is the decomposition for of $F_{N}(w)$ and *f.*

So, we can get the force of the centrifugal slider in the X and Y direction

(4) {Fx=F−Fx′−Ffx=F−FN(w)(cos(α2)+μsin(α2))Fy=Fy′−Ffy=FN(w)(sin(α2)−μcos(α2))

### 2.3. Deflection Calculation

**Figure 6:**
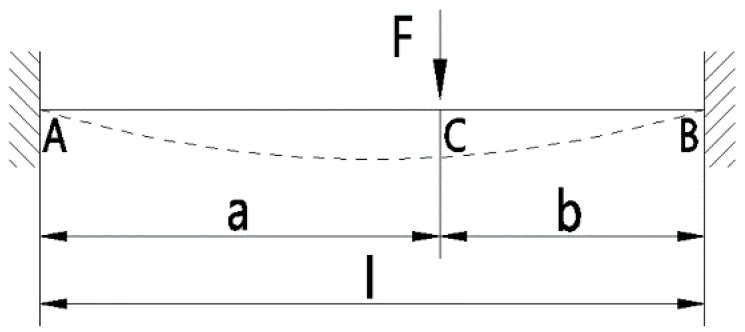
The schematic diagram of elastic-beam deflection analysis.

According to the cantilever deflection equation of applied engineering mechanics, the Figure 6 can be obtained.

According to the Figure 6, we can get the deflection

(5) $$w=\{\begin{array}{ll} -\frac{Fb^{2}x^{2}}{6EIl}[3\frac{a}{l}-(1+2\frac{a}{l})\frac{x}{l}] & \left(0\leq x\leq a\right)\\ -\frac{Fa^{2}{\left(l-x\right)}^{2}}{6EIl}[\frac{a}{l}-(1+2\frac{b}{l})\frac{\left(l-x\right)}{l}] & \left(a<x\leq l\right) \end{array}$$

According to Figure 4 and Figure 6, we can get

(6) F=Fy; b=L−L′4; a=L+5L′4; l=2L+L′; I=HB312

where, *H* is the thickness of the elastic-beam.

If $F_{x}=0$ in (4), the maximum value of $F_{N}(w)$ can be obtained

(7) FNmax(w)=F[cos(α2)+μsin(α2)]−1

According to the (5)–(7), the maximum deflection at node *C* can be obtain.

(8) wCmax=4F(L−L′4)3(L+5L′4)3(sin(α2)−μcos(α2))[EHB3(2L+L′)3(cos(α2)+μsin(α2))]−1

Combined with Figure 4, the stroke of the centrifugal slider can be obtained

(9) S=wCmaxtan(α2)

### 2.4. Kinematic Analysis and Time Estimating

**Figure 7:**
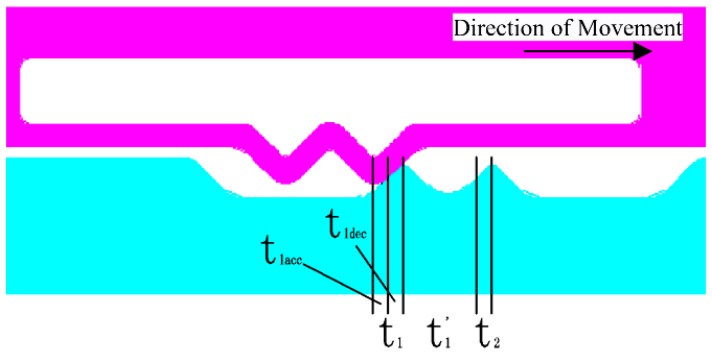
The schematic diagram of kinematic analysis and time estimating.

The schematic diagram of kinematic analysis and time estimating are shown in Figure 7.

#### 2.4.1. The First Contact of Active Tooth to Passive Tooth

The first contact is divided into two stages, one stage (*t*_1*acc*_) is the uniformly acceleration process when the active tooth from the initial position to the first contact; the other stage (*t*_1*dec*_) is the uniform deceleration process when the active tooth from the first contact to separation.

And

(10) $$S=v_{0}t+\frac{1}{2}at^{2}$$

where, *S* is the displacement of the active tooth; *v*_0_ is the initial velocity of the active tooth; *a* is the average acceleration of the active tooth; *t* is the time of the whole movement process.

• The uniformly acceleration process

We can get

(11) {v1=0a1=FmS1=Etan(α2)

where, *v*_1_ is the initial velocity of the active tooth at first contact; *a*_1_ is the average acceleration of the active tooth at first contact; *S*_1_ is the displacement of the active tooth from the initial position to the first contact.

So

(12) t1acc=2Etan(α2)r0(2πn60)2

where, *r*_0_ is the initial eccentricity of centrifugal slider.

• The uniformly deceleration process

The uniform deceleration process is the inverse of the uniform acceleration process.

So

(13) t1=t1acc+t1dec=2t1acc=22Etan(α2)r0(2πn60)2

#### 2.4.2. The Second Contact of Active Tooth to Passive Tooth

The second contact is divided into two stages, one stage (*t*_1_*’*) is the uniformly acceleration process when the active tooth from separation to second contact; other stage (*t*_2_) is the uniform deceleration process when the active tooth from the second contact to separation.

• The uniformly acceleration process

We can get

(14) {v1′=0a1′=(r0+L′2)+(r0+L′2+L″−E⋅tan(α2))2(2πn60)2v2=2a1′(L″−E⋅tan(α2))+v1′2

where, *v*_1_*’* is the velocity of the active tooth separation from the first passive tooth; *a*_1_*’* is the average acceleration of the active tooth from separation to second contact; *v*_2_ is the velocity of the active tooth at second contact

So

(15) $$t_{1}{}^{\prime }=\frac{v_{2}-v_{1}{}^{\prime }}{a_{1}{}^{\prime }}$$

• The uniformly deceleration process

The uniform deceleration process is the inverse of the uniform acceleration process.

We can get

(16) a2=(r0+L′2+L″−E⋅tan(α2))+(r0+L′2+L″)−2r02(2πn60)2=L′+2L″−E⋅tan(α2)2(2πn60)2

where, *a*_2_ is the average acceleration of the active tooth the second contact to separation.

So

(17) E⋅tan(α2)=v2t2+12a¯2t22

According to the (14), (16), and (17), we can get the time *t*_2_.

According to the above theoretical analysis, the maximum deflection and time estimating of the different parameterized variable can be obtained, shown as Table 2.

## 3. Simulation Analysis

**Figure 8:**
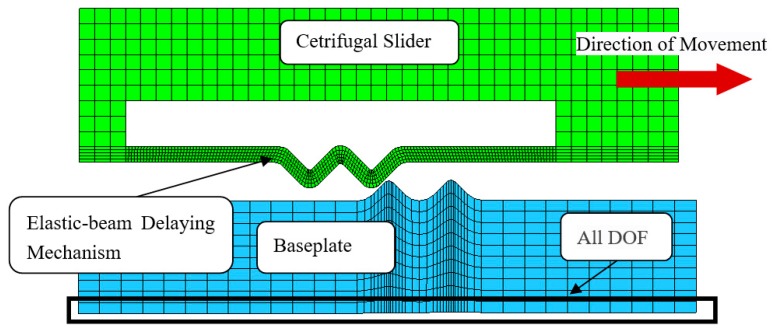
The infinite model of elastic-beam delaying mechanism.

**Figure 9:**
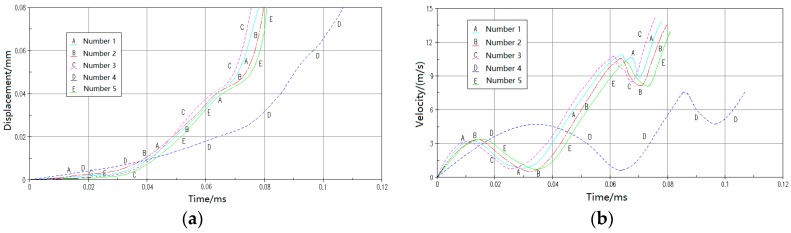
The simulation results of different structural parameters (**a**) displacement-time curves (**b**) velocity -time curves.

Base on the Figure 4 and Table 1, establishing the infinite model of elastic-beam delaying mechanism (Figure 8) by using HyperMesh, applying Material properties (Table 3), loading the acceleration of a constant 30,000 g, and making nonlinear dynamic mechanics simulation by using ANSYS/LS-DYNA, displacement-time curves (Figure 9a) and velocity-time curves (Figure 9b) are obtained.

The Table 4 shown that the simulation results of the deflection are in good agreement with the theoretical results, and the maximum error is less than 6.5%, because of the residual velocity of separation between active teeth and passive when simulating.

According to Table 2, Table 4 and Figure 9, the following conclusions are obtained:

- In terms of movement trend, the simulation results are in good agreement with the theoretical calculation;
- In terms of the movement time for the same displacement, simulation results are shorter than the theoretical calculation. Because when active tooth is separated from the first passive tooth, the simulation results have residual velocity, and the theoretical value is 0;
- The gap between active tooth and passive tooth is the most important factor affecting the movement time.

## 4. Fabrication

**Figure 10:**
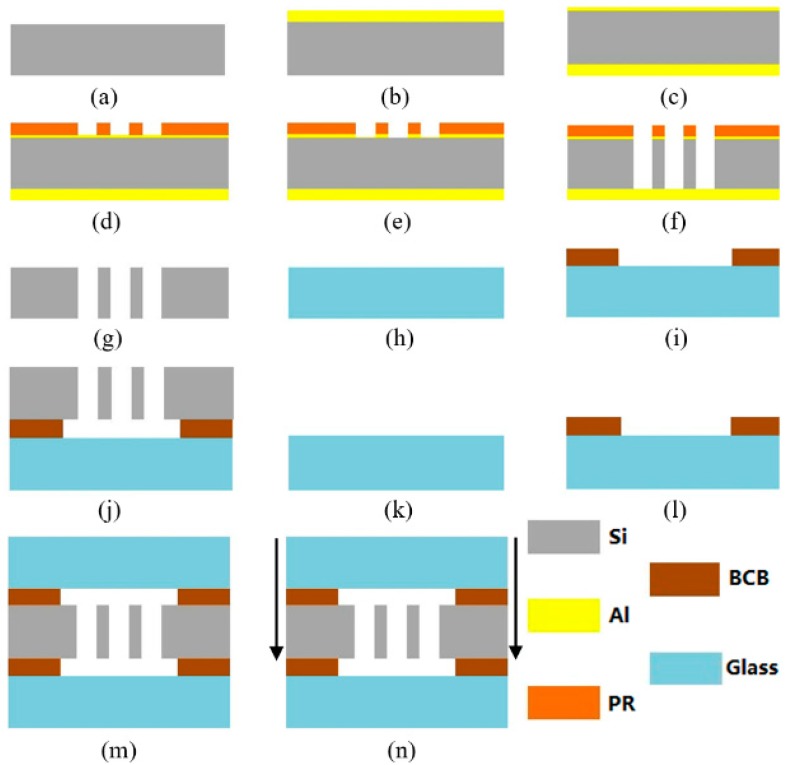
Micromachining process of silicon-based MEMS S&A system (**a**) Back-up; (**b**) Physical Vapor Deposition (PVD) Al 2um; (**c**) PVD Al 0.2um; (**d**) Structural lithography; (**e**) Corroded aluminum; (**f**) Deep Reactive Ion Etching (DRIE); (**g**) Degumming; (**h**) Back-up; (**i**) BCB Lithography; (**j**) BCB bond; (**k**) Buck-up; (**l**) BCB Lithography; (**m**) BCB bond; (**n**) Scribing.

**Figure 11:**
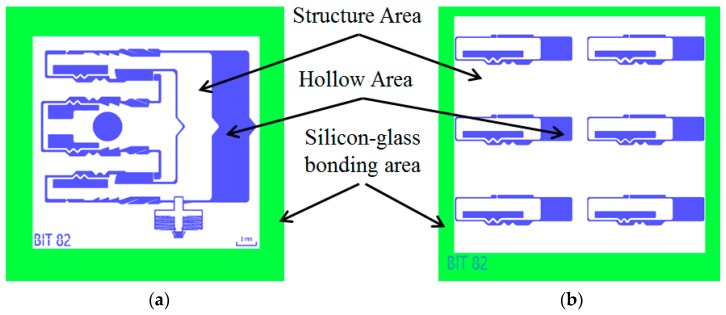
The processing layout (**a**) MEMS S&A system, (**b**) Elastic-beam delaying mechanism.

The Figure 10 shows the micromachining process of silicon-based MEMS S&A system. It mainly includes the silicon-based MEMS S&A system in the middle and Benzocyclobutene (BCB) bonded glass on the upper and lower sides. As shown in Figure 11, the white part is the structure area, which is the main structure left by photolithography with bulk-micromachining technology, the blue part is the hollow area, the green part is the silicon-glass bonding area with BCB bonding, which enables the structure area to generate a certain gap, so that the key structure can move along the predetermined mode in the hollow area.

**Figure 12:**
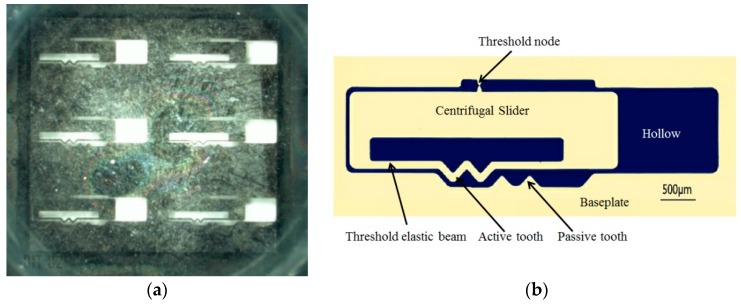
The micro-sample of the elastic-beam delaying mechanism (**a**) overall view, (**b**) partial view.

The micro-sample of the elastic-beam delaying mechanism can be obtained based on the digital microscope VHX-6000 series produced by Keyence Corporation, as shown in Figure 12a. And partial view can be obtained based on the Electron microscope, as shown in Figure 12b.

## 5. Test

**Figure 13:**
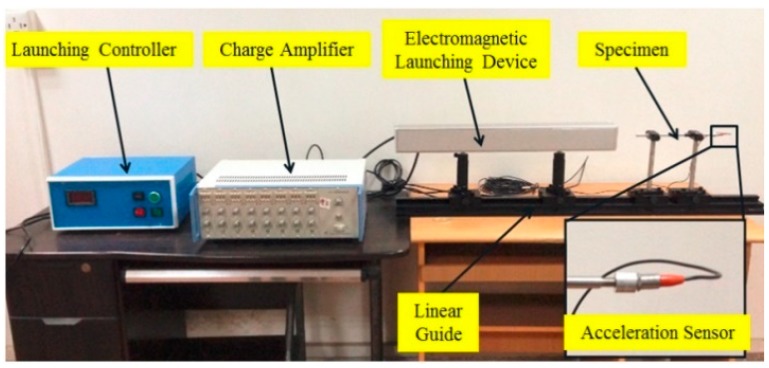
Impact test platform.

**Figure 14:**
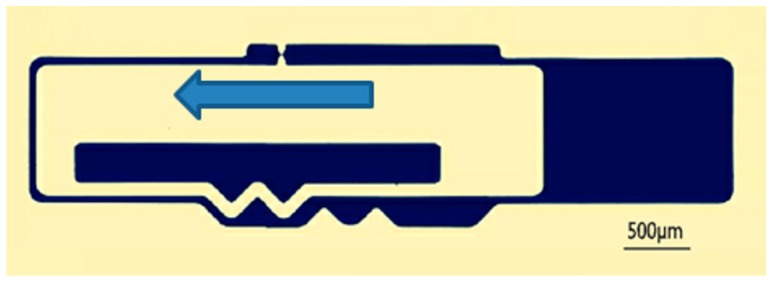
The direction of impact acceleration.

**Figure 15:**
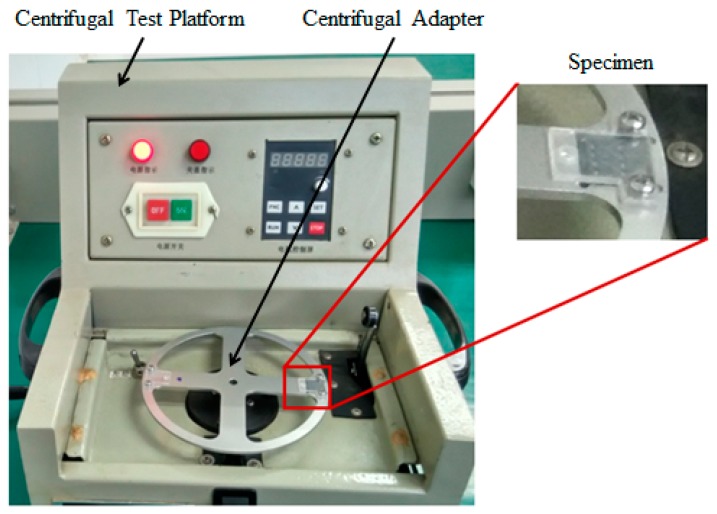
Centrifugal test platform and centrifugal adapter.

**Figure 16:**
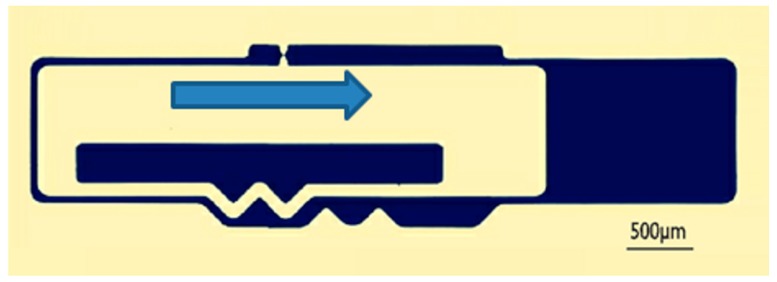
The direction of centrifugal acceleration.

There are two kinds of tests for delaying mechanism:

- The impact test. The aim is to break the threshold node so as to ensure the centrifugal test. The impact test platform is shown in Figure 13, and impact acceleration direction is shown in Figure 14.
- The centrifugal test. The aim is to obtain g-value of the centrifugal slider moving through the passive tooth of the delaying mechanism with different parameters. The centrifugal test platform is shown in Figure 15, and centrifugal acceleration direction is shown in Figure 16.

**Figure 17:**
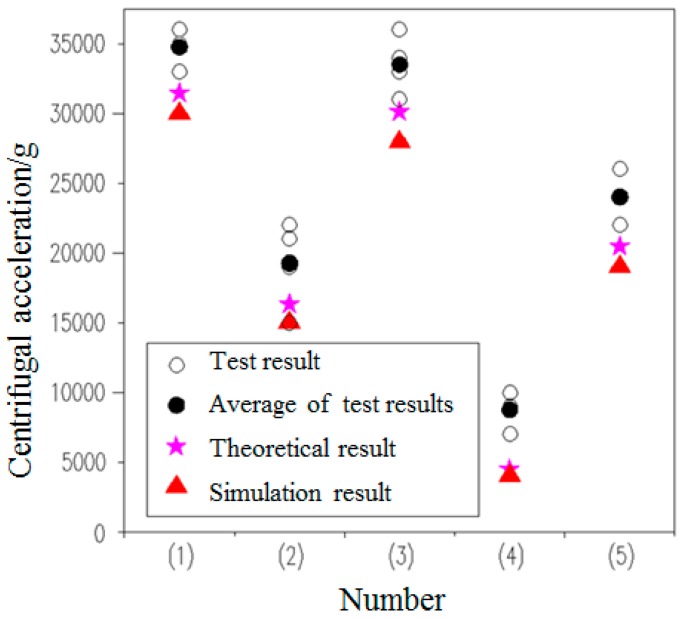
The g-value of the delaying mechanism with different parameters.

Four groups of centrifugal tests were carried out on the parameterized delaying mechanism, as shown in Table 1. The theoretical and simulation results obtained from Formula (8) were combined with centrifugal test to obtain Table 5 and Figure 17.

According to Table 5 and Figure 17, it arrives at the conclusions as follows:

- The g-value of the delaying mechanism with the same processing batch and the same parameter is relatively discrete, indicating that the material properties of silicon have a certain degree of dispersion.
- The theoretical results are all higher than the simulation results, because the theoretical calculation is completely static, and the possible initial velocity is ignored. And the average value of the test results is higher than the theoretical results, because of the friction and gas resistance in the micro-sample.
- Theoretical results, simulation results and test results have a high degree of agreement, which can be used for initial optimization design.

## 6. Conclusions

In this paper, the elastic-beam delaying mechanism has been proposed innovatively. Combining with the rigid dynamic mechanics theory, the mathematical model was established. Simulation and test results match theoretical results quite well. It is believed that the elastic-beam delaying mechanism is quite effective and useful to slow the speed of the movable part in MEMS devices.

## Figures and Tables

**Table 1:** The parameters of elastic-beam delaying mechanism.

Number	1	2	3	4	5
Width of the elastic-beam ***B*/mm**	0.1	0.08	0.1	0.1	0.1
Angle of active tooth *α*/°	90	90	70	90	90
Length of the elastic-beam ***L*/mm**	1.2	1.2	1.2	2.4	1.4
Gap between active and tooth and passive tooth ***E*/mm**	0.03	0.03	0.02	0.03	0.03
Number of the active tooth	2				1
Distance between two adjacent active teeth ***L*′/mm**	0.4				-
Distance between two adjacent passive teeth ***L*″/mm**	0.4				

**Table 2:** The theoretical result of maximum deflection and time estimating.

Number		1	2	3	4	5
Mass of centrifugal slider ***m***/kg		2.88 × 10^−6^	2.836 × 10^−6^	2.866 × 10^−6^	2.84 × 10^−6^	2.868 × 10^−6^
Maximum deflection ***w_max_***/mm		2.86 × 10^−2^	5.50 × 10^−2^	2.0 × 10^−2^	0.20	3.23 × 10^−2^
Time estimating/μs	*t*_1_	27.6	28.3	23.1	73.0	29.3
	*t*_1_′	49.7	48.0	50.3	36.5	49.5
	*t*_2_	1.9	3.8	1.3	18.3	2.2
	*t*_total_	79.2	80.1	74.7	129.8	81

**Table 3:** The material properties [21].

Name	Density ρ (kg/m^3^)	Elasticity Modulus *E* (GPa)	Poisson’s Ratio ν
Si	2.3 × 10^3^	180	0.3

**Table 4:** The maximum deformation and error between theoretical results and simulation results.

Number		1	2	3	4	5
Maximum deflection ***w_max_***/mm	Theoretical	2.86 × 10^−2^	5.50 × 10^−2^	2.0 × 10^−2^	0.20	3.23 × 10^−2^
	Simulation	2.92 × 10^−2^	5.64 × 10^−2^	2.1 × 10^−2^	0.213	3.37 × 10^−2^
Error (Simulation-Theoretical)/Theoretical		2.1%	2.5%	5.0%	6.5%	4.3%

**Table 5:** The g-value of the delaying mechanism with different parameters.

Number	Test Results/g					Theoretical Results/g	Simulation Results/g
	Group 1	Group 2	Group 3	Group 4	Average		
(1)	35,000	33,000	35,000	36,000	34,750	31,500	30,000
(2)	19,000	15,000	21000	22,000	19,250	16,370	15,000
(3)	33,000	31,000	34,000	36,000	33,500	30,120	28,000
(4)	9000	7000	10,000	9000	8750	4520	4100
(5)	24,000	22,000	24,000	26,000	24,000	20,500	19,000
